# The usefulness of Integrative Neuromuscular Training to counteract obesity: a narrative review

**DOI:** 10.1038/s41366-023-01392-4

**Published:** 2023-09-30

**Authors:** Luca Cavaggioni, Luisa Gilardini, Marina Croci, Damiano Formenti, Giampiero Merati, Simona Bertoli

**Affiliations:** 1 Obesity Unit - Laboratory of Nutrition and Obesity Research, Department of Endocrine and Metabolic Diseases, IRCCS Istituto Auxologico Italiano, 20145 Milan, Italy; 2 Department of Biotechnology and Life Sciences, University of Insubria, 21100 Varese, Italy; 3 IRCCS Fondazione don Carlo Gnocchi, 20148 Milan, Italy; 4 International Center for the Assessment of Nutritional Status (ICANS), Department of Food, Environmental and Nutritional Sciences (DeFENS), University of Milan, 20133 Milan, Italy

**Keywords:** Obesity, Weight management

## Abstract

**Background and objective:**

The association between physical activity and diet has a valuable impact in weight status management to counteract obesity. In this context, within different training strategies (i.e., endurance, resistance training, concurrent training, agility training) the Integrative Neuromuscular Training (INT) represents a structured training mode focused on global human movement pattern development with the aim to enhance motor control, mobility and stability. In this narrative review we aimed to discuss the feasibility of INT interventions on physical fitness and body composition outcomes in individuals with obesity.

**Subjects:**

Medline/PubMed, EMBASE, Web of Science, Google Scholar including were searched before 1^st^ February 2023 without restrictions on publication year.

**Methods:**

Two researchers extracted data from published trials. Randomized controlled trials or clinical trials, Body Mass Index of children and adolescents at the 95% percentile or greater, and for adults to be above 30 kg/m^2^, detailed intervention description, randomization process and allocation into an experimental or a control group, trials must have been written in English, were included.

**Results:**

We included a total of 19 studies complying with the inclusion criteria for the review process. There is evidence that INT promotes positive adaptations in fitness levels in both younger and older participants with concomitant ameliorations during a shorter, medium and longer time period. Moreover, cardiorespiratory fitness, muscular strength, balance, postural control and body composition reached significant remarkable improvements following a specific intervention based on INT principles compared to other training mode. However, Body Mass Index, fat mass percentage and waist circumference showed similar changes overtime.

**Conclusions:**

Taken together, these findings support the effectiveness of INT in ameliorating physical fitness (i.e., health-related and skill related components) without negative changes in body composition. Nevertheless, fitness coaches and therapists may consider this training modality a feasible option when prescribing physical exercise in outpatients with obesity.

## Introduction

Obesity represents a remarkable health metabolic disease characterized by an increased body fat that could lead to an augmented risk of cardiovascular disease, diabetes mellitus, hypertension and mortality [1, 2]. An adequate nutrition strategy, psychological-behavioral support as well as physical activity represent key factors in weight status management [3, 4]. Several international guidelines, such as the World Health Organization (WHO) and the American College of Sports Medicine (ACSM) recommend to respect an amount of 150–250 min per week of moderate-intensity physical activity to obtain a significant weight loss [5–7].

In this context, physical fitness refers to the body’s ability to perform daily-life activities efficiently, which is determined by different components, including “health-related” factors, such as cardiorespiratory fitness, muscular strength and endurance, flexibility, and “skill-related” variables as agility, motor coordination, balance, power, reaction time and speed [7, 8].

Cardiorespiratory fitness can be improved through various form of continuous or intermittent exercises with the aim to improve cardiorespiratory function [6, 9]. For people with obesity, it is particularly suggested to observe an exercise intensity between 49% and 85% of peak aerobic capacity to induce several modifications in visceral adipose tissue [10] and body fat reduction [11].

For what concern muscular strength, the purpose is to increase skeletal muscle mass involving major muscle groups [7]. Strength training for obesity might be helpful to improve myogenesis, lean mass and protein synthesis, characteristics particularly suited to counteract sarcopenic obesity [12]. Additionally, the combination between aerobic and strength training modalities (known as Concurrent Training) seems to be effective in improving body composition, cardiorespiratory fitness and muscular strength [11, 13] with little interference on muscle size growth [14]. Individual with obesity may also obtain other positive effects by following a dietary restriction program [11, 15].

For flexibility, obesity may impair joint range of motion because the adipose tissue interposed around each joint may limit segmental body rotation, causing mechanical interference [16], especially in elbow, hip and knee segments [17].

Lastly, when dealing with skill-related components of physical fitness, especially balance and motor coordination, individuals with obesity exhibit a reduced medio-lateral or sagittal stability and motor coordination with a concomitant center of pressure anteriorly shifted, due to compensatory motions counteracting body weight accumulation [18, 19].

Traditionally, all of these physical fitness’ components can be improved through specific movements or exercises targeting a single specific outcome (i.e., aerobic, strength, balance, flexibility) in an isolated mode during an entire training session.

An innovative training regimen that embraces the interaction between all of each component is called *Integrative Neuromuscular Training* (INT) [20]. This modality can be a suitable option to target the overall physical fitness (health and skill-related variables) in an integrated manner. INT is a training approach targeted on global human movement pattern development (e.g., fundamental movement skills) and specific strength and conditioning exercises (e.g., motor control, mobility, strength, proprioception, cardiorespiratory fitness), with the aim to restore a correct movement mechanics by stressing an efficient quality of movement and physical fitness [21, 22].

Following INT principles, improvements in muscular strength, power, motor skill performance, dynamic stability and balance [21, 23] were observed in children and adolescents [24–26]. Additionally there is an enhancement in motor competence and proficiency [27], as well as a reduction in movement asymmetries and “motor awkwardness”[28]. Moreover, a recent systematic review reported that INT is an effective training mode to improve motor performance and injury prevention especially in young athletes [29]. Taken together, these findings provide evidence supporting INT as a training mode to target the overall physical fitness by focusing on the quality of movement.

To the best of the Authors’ knowledge, the existing literature regarding the effectiveness of INT on individuals with a noteworthy health condition (i.e., obesity) is scarce. However, from a theoretical point of view, INT could be helpful for ameliorating fitness levels in people with obesity thanks to a mixture of stimuli within the same training session.

Therefore, the aim of this narrative review was to identify and synthesize the feasibility of INT intervention on health-related, skill-related and body composition outcomes in individuals with obesity.

## Methods

### Data sources and searches

This narrative review was structured with a computerized search of four electronic databases (Medline/PubMed, EMBASE, Web of Science, Google Scholar) including articles published before 1^st^ February 2023 without restrictions on publication year. Articles were limited to human experiments. The search used a combination of the following terms “integrative neuromuscular training” OR “integrative neuromuscular exercise” OR “integrative neuromotor training” OR “integrative neuromotor exercise” OR “neuromotor training” OR “neuromotor exercise” OR “neuromuscular training” OR “neuromuscular exercise” OR “balance training” OR “balance exercise” OR “proprioception training” OR “proprioception exercise” OR “postural control” OR “postural stability” OR “cognitive training” OR “functional training” OR “functional movement” OR “quality of movement” OR “movement quality” AND “obes*“.

### Study selection

In order to exclude duplicate studies, two authors (L.C. and D.F.) reviewed the abstracts of each study respecting the following inclusion criteria: a) randomized controlled trials or clinical trials, b) Body Mass Index (BMI) of children and adolescents at the 95% percentile or greater, and for adults to be above 30 kg/m^2^, c) detailed intervention description (an health-related component associated with, at least, one skill-related outcome), d) randomization process and allocation into an experimental or a control group, e) trials must have been written in English and published in a peer-reviewed journal. Studies with participants with severe cardiovascular, neurological or physical comorbidities, co-intervention (psychological or medical support) or duration less than 3 weeks were excluded.

### Data extraction

Two authors (L.C., D.F.) independently screened the articles by using title and abstracts. Abstracts were excluded based on the exclusion criteria. Next, from the initial list, the full text of the selected articles was screened by those two authors to verify whether they met the inclusion criteria excluding duplicate studies. Finally, they compiled a list of all eligible articles for this review. It is worth emphasizing that the participants’ ages, gender, race/ethnicity, etc., were not restricted in order to obtain a more complete understanding of this topic. The studies were considered when a consensus was reached by two authors (L.C., D.F.) for a later presentation and discussion. If in presence of a disagreement between these two authors, a third researcher (L.G.) made the definitive decision.

## Results

The review procedure generated 147 papers from three electronic databases and after an initial removal before screening (e.g., duplicate articles), a total of 19 studies complying with the inclusion criteria were considered eligible for the review process. The overall number of participants was 828, with an age range from 7 to 70 years with a comprehensive intervention’s duration that ranges from three weeks up to twelve months (Table 1).

**Table 1:** Characteristics of included studies.

Reference	Study duration	Sample size and age	Number of groups	Exercise intervention based on INT principles	Outcome measurements	Results	INT components’ adherence
Guzmán-Muñoz et al. (2019)	4 weeks	32 children (7–9 years)	2 EG § CG	2 times/week of exercises focused on lower limbs muscular strength and coordination: (i) mini hurdle jumping, (ii) agility cone drills, and (iii) agility ladder drills. Then, exercises to improve balance: (i) single-leg balance, (ii) marching in place, (iii) tandem walk, and (iv) single-leg squat progressing from stable surface with eyes open to an unstable surface with eyes closed	Static postural control detecting center of pressure COP variables (sway area, mean velocity, Antero-Posterior velocity, Medio-Lateral velocity) Dynamic postural control with Star Excursion Balance Test SEBT (anterior reach, postero-lateral reach, postero-medial reach)	(+) in COP mean velocity, sway area (+) in SEBT anterior reach, postero-lateral reach, postero-medial reach	Partial (Strength, motor coordination, agility, balance)
Batrakoulis et al. (2018)	40 weeks	49 adult women (36.4 ± 4.4 years)	3 EG § EG with detraining § CG	3 times/week of high-intensity circuit-type neuromuscular exercise training with the use of asynchronous music incorporating fundamental movement patterns using bodyweight exercises using portable modalities (suspension, belts, balance balls, kettlebells, medicine balls, battle ropes, stability balls, speed ladders, foam rollers, elastic bands)	Muscular strength (one repetition maximum leg press) Cardiorespiratory fitness (VO2_max_ on treadmill) Daily energy expenditure (portable indirect calorimetry) Body composition (body mass, body mass index, lean body mass, waist circumference, resting metabolic rate)	(+) in muscular strength (+) in cardiorespiratory fitness (+) in daily energy expenditure (+) in body composition	Complete (strength, stability, agility, motor coordination, cardiorespiratory fitness, agility, mobility)
Batrakoulis et al. (2021)	10 months	49 inactive adult women (36.4 ± 4.4 years)	3 EG § EG with detraining § CG	3 times/week of progressive loaded fundamental movement patterns circuit training (squat, hinge, lunge, push, pull, carry, rotation, plank) using portable modalities (suspension, belts, balance balls, kettlebells, medicine balls, battle ropes, stability balls, speed ladders, foam rollers, elastic bands) with bodyweight exercises	Total body bone mineral density (BMD) and total bone mineral content (BMC) with dual-energy X-ray absorptiometry Flexibility (passive joint range of motion in ankle dorsiflexion, knee extension, hip extension, shoulder extension, glenohumeral internal rotation) Static balance (Romberg test) Functional performance (Functional Movement Screen) Muscular strength (one repetition maximum vertical chest press, supinated closed-grip lat pull-down, leg extension, lying leg curl Local muscular endurance (maximum number of repetitions in 60 s in partial curl-up, kneeling push up, chair squat)	(+) in BMD (+) in BMC (+) in muscular strength and local muscular endurance, (+) in flexibility (+) in static balance	Complete (strength, stability, agility, motor coordination, cardiorespiratory fitness, agility, mobility)
Batrakoulis et al. (2020)	10 months	49 inactive adult women (36.4 ± 4.4 years)	3 EG § EG with detraining § CG	3 times/week of supervised protocol characterized by a hybrid format including a mix of endurance training, core strengthening and resistance training elements with the use of asynchronous music incorporating fundamental movement patterns using bodyweight exercises using portable modalities	Psychosocial distress (General Health Questionnaire, GHQ-12) Subjective vitality (subjective vitality scale, SVS) Exercise behavioral regulation (Behavioral Regulation in Exercise Questionnaire–2, BREQ-2)	(+) in psychological distress (+) in subjective vitality (+) in exercise behavioral regulation	Complete (strength, stability, agility, motor coordination, cardiorespiratory fitness, agility, mobility)
Batrakoulis et al. (2022)	12 months	97 adult women (44.8 ± 5.2 years)	4 EG 1day/week § EG 2days/wee§ EG 3days/wee§ CG	Progressive loaded fundamental movement patterns circuit training regimen integrating low-to-moderate impact cardiovascular bodyweight drills and compound resistance training exercises performed intermittently. Each session used 6–12 whole-body exercises and work-to-rest ratios ranging from 1:3 to 2:1 with a duration of 15–45 seconds	Flexibility (passive joint range of motion in modified sit&reach, ankle dorsiflexion, knee flexion, hip flexion, shoulder flexion) Static balance (Romberg test) Functional performance (Functional Movement Screen) Muscular strength (one repetition maximum vertical chest press, supinated closed-grip lat pull-down, leg extension, lying leg curl Local muscular endurance (maximum number of repetitions in 60 s in partial curl-up, kneeling push up, chair squat)	(+) in muscular strength and local muscular endurance especially in EG 3days/week (+) in flexibility especially in EG 3days/week (+) in static balance especially in EG 3days/week (+) in functional performance especially in EG 3days/week	Complete (strength, stability, agility, motor coordination, cardiorespiratory fitness, agility, mobility)
Molina-Garcia et al. (2019)	13 weeks	70 children (10.8 ± 1.2 years)	2 EG § CG	3 times/week of movement-quality exercise training composed by self-awareness of analytical movement patterns (e.g., anterior and posterior pelvic tilt), body posture (e.g., optimal spine position), mobility (e.g., hip flexion mobility), stability (e.g., core stability), strength (e.g., bilateral lower limb push strength, (e.g., squat pattern) combined with “multi-games” aerobic exercise from moderate-to-vigorous intensity	Plantar pressure during walking for 10 times along a 10 m-long corridor on a pressure platform (plantar surface area, plantar maximum force, force-time integrals)	(=) in plantar pressure surface area (=) in plantar force time integrals (+) in plantar maximum force	Complete (strength, stability, agility, motor coordination, cardiorespiratory fitness, agility, mobility)
Molina-Garcia et al. (2020)	13 weeks	64 children (10.9 ± 1.3 years)	2 EG § CG	3 times/week of movement-quality exercise training composed by self-awareness of analytical movement patterns (e.g., anterior and posterior pelvic tilt), body posture (e.g., optimal spine position), mobility (e.g., hip flexion mobility), stability (e.g., core stability), strength (e.g., bilateral lower limb push strength, (e.g., squat pattern) combined with “multi-games” aerobic exercise from moderate-to-vigorous intensity	Body posture (2-dimensional photogrammetry measuring lower limb angle and plumb-tragus distance in sagittal and frontal plane) Functional performance (Functional Movement Screen) Muscular strength (one repetition maximum arm press, leg press, handgrip strength test, standing long jump) Cardiorespiratory fitness (20 meters shuttle run test and agility 4×10 shuttle run test)	(+) in lower limb angle and plumb-tragus distance in sagittal plane (+) in lower limb angle in frontal plane (+) in functional performance (+) in one repetition maximum arm press, in handgrip strength test, standing long jump (=) in cardiorespiratory fitness	Complete (strength, stability, agility, motor coordination, cardiorespiratory fitness, agility, mobility)
Molina-Garcia et al. (2022)	13 weeks	50 children (10.7 ± 1.2 years)	2 EG § CG	3 times/week of movement-quality exercise training composed by self-awareness of analytical movement patterns (e.g., anterior and posterior pelvic tilt), body posture (e.g., optimal spine position), mobility (e.g., hip flexion mobility), stability (e.g., core stability), strength (e.g., bilateral lower limb push strength, (e.g., squat pattern) combined with “multi-games” aerobic exercise from moderate-to-vigorous intensity	Biomechanics during walking 7 gait cycles with motion capture analysis (cadence, stance/support times, step length, stride width pelvis/hip/knee/ankle kinematics in three planes) Muscoskeletal pain (Pediatric Pain Questionnaire)	(+) in stance time (+) in foot abduction (+) in pelvic tilt (=) in cadence, step length, stride width, knee/hip/ankle kinematics (=) in muscoskeletal pain	Complete (strength, stability, agility, motor coordination, cardiorespiratory fitness, agility, mobility)
Cavaggioni et al. (2019)	6 weeks	64 adults (50.5 ± 10.4 years)	2 EG § CG	3 times/week of movement-quality exercise training emphasizing mobility, stability and neuromuscular training. t was based on self-awareness about the movement execution while performing multi-joint strength exercises, diaphragmatic breathing and corrective postures to emphasize motor control in a circuit training mode	Functional performance (Functional Movement Screen) Static balance (Modified Balance Error Scoring System) Muscular strength (handgrip strength test, five repetition sit-to-stand test) Breathing pattern (total faulty breathing scale) Body composition (fat mass %, waist circumference, body mass index)	(+) in functional performance (+) in static balance (=) in muscular strength (+) in breathing pattern (=) in body composition	Partial (stability, mobility, strength, cardiorespiratory fitness)
Alizadeh et al. (2022)	8 weeks	25 adult women (26.3 ± 4.7 years)	3 EG active individuals § EG inactive individuals § CG	3 times/week of integrated resistance training including upper and lower limbs multi-joint movements, core stability, motor coordination and balance exercise in a circuit training mode	Cognitive-psychological performance, brain-derived neurotrophic factor (BDNF) Cognitive-psychological performance, executive function (number of errors, number of true responses, reaction time)	(+) Brain-derived neurotrophic factor (+) Executive function	Partial (strength, balance, motor coordination, stability)
Santanasto et al. (2015)	12 months	36 adults (70.6 ± 6.1 years)	2 EG § CG	3 times/week of physical activity combined with weight loss program composed by treadmill walking, strength training with ankle weight (standing leg curl, knee extension, side hip raise and a toe stand) and balance exercises	Functional performance (Short Physical Performance Battery) Body composition (visceral fat, percent body fat, intermuscular adipose tissue, subcutaneous tight, muscle density, waist circumference, body weight, body mass index	(+) in short physical performance battery (+) body composition (in visceral fat, percent body fat, intermuscular adipose tissue, subcutaneous tight, body weight, body mass index) (=) muscle density, waist circumference	Partial (cardiorespiratory fitness, strength, balance)
Bezzoli et al. (2018)	3 weeks	20 adults (52.7 ± 9 years)	2 EG § CG	5 times/week of 30-min conditioning session on a bicycle ergometer at an intensity of 60–70% of heart rate maximum combined with resistance training exercises in a circuit mode with the addition of motor control exercises to improve postural alignment, intra-abdominal pressure, rib cage mobility, and the perception of correct muscle activation of transversus abdominis, diaphragm, internal oblique, and pelvic floor muscles	Pulmonary function with spirometry (Forced Vital Capacity, Forced Expiratory Volume 1 second, Maximum Voluntary Ventilation) Respiratory muscles strength (maximal inspiratory pressure, maximal expiratory pressure Thoracic excursion using a 200-cm tape Cardiorespiratory fitness (6 minute walking test) Quality of life (Short Form Health Survey questionnaire, SF36) Body composition (fat mass, fat free mass, body mass index)	(+) in pulmonary function, respiratory muscles strength, thoracic excursion (=) in cardiorespiratory fitness, body composition and quality of life	Paartial (balance, stability, cardiorespiratory fitness)
Bonney et al. (2019)	14 weeks	52 adolescents (13-16 years)	EG § CG	1 time/week in task-orientated training protocol consisting 3 components including dance with music, goal-directed activities (e.g., throwing at targets, emptying boxes, walking, and running), and team-based games (e.g., tag, relays, hide-and-seek).	Cardiorespiratory fitness (20 meters shuttle run test) Motor coordination (Movement Assessment Battery for Children test 2nd edition) Muscular strength (lower extremity hand-held dynamometer, standing long jump, sit to stand, lateral step-up, stair climbing) Anaerobic fitness (10 × 5 meters sprint test) Cognitive-psychological performance (self-efficacy questionnaire, Children’s Self-perceptions of Adequacy in and Predilection for Physical Activity)	(=) in cardiorespiratory fitness, muscular strength, motor coordination and anaerobic fitness (−) cognitive performance	Partial (cardiorespiratory fitness, motor coordination, balance, agility)
Rojhani-Shirazi et al. (2016)	4 weeks	32 adults (36.1 ± 6 years)	2 EG § CG	4 times/week of physical activity combined with balance training exercises, standing on one leg, standing in tandem mode, standing on one leg with closed, walking in a tandem mode, walking on toes and heels, side walk, standing while one upper extremity and the opposite lower extremity were up, rotating the head from side to side, walking backwards for four steps, and shifting weight from one foot to the other	Dynamic postural control with Star Excursion Balance Test SEBT (anterior reach, postero-lateral reach, postero-medial reach) Static postural control with Single Leg Stance (SLS) Quickness and agility with Get up and Go (GUG)	(+) in static and dynamic postural control (+) in quickness and agility	Partial (balance, stability)
Rodrigues-Cambiriba et al. (2021)	8 weeks	59 adults (40-59 years)	2 EG § CG	3 times/week of functional training (half squat with fitball between thigths, standing hip, squatting laterally with bend performing leg abduction, walking and jogging, TRX row, pull rope, push up with knees, lumberjack movement) combined with dietary education	Cardiorespiratory fitness (6 minute walking test) Muscular strength (handgrip test, maximum isometric lumbar traction) Local muscular endurance (60 second abdominal test) Flexibility (Wells bench test) Physical activity level (International Physical Activity Questionnaire) Quality of life (Medical Outcomes Study 12) Knee pain (WOMAC) Body composition (body fat, body fat percentage, skeletal mass, body mass index)	(+) in muscular strength test (maximum isometric lumbar traction) (−) cardiorespiratory fitness, muscular strength (handgrip strength), local muscular endurance, flexibility, quality of life, knee pain, physical activity level, body composition	Partial (cardiorespiratory fitness, strength, motor coordination)
Maffiuletti et al. (2005)	3 weeks	39 adults (20-40 years)	3 EG § EG CG	5 times/week of 30-min conditioning session on a bicycle ergometer at an intensity of 60–70% of heart rate maximum combined with resistance training exercises (40-70% of one repetition maximum load on lower and upper-body machines) with the addition of proprioceptive exercises using a movable platform (Delos system) in a single leg stance	Static postural control (time of balance maintenance in single leg stance, mean errors on medial-lateral direction, mean errors of trunk motion)	(+) in static postural control	Partial (balance, stability, cardiorespiratory fitness)
La Scala Teixeira et al. (2020)	30 weeks	44 adults (39.7 ± 5 years)	3 EG § EG CG	3 times/week of aerobic exercises performed on an ergometer combined with resistance training performed in a circuit mode (squat thruster, hip flexion with elbow flexion, ball crunch, side lateral raise with lunge, horizontal row, stiff leg deadlift, bench press, single leg balance eyes closed)	Cardiorespiratory fitness (VO2max on treadmill) Physical activity level (International Physical Activity Questionnaire) Body composition (fat mass percentage, lean muscle mass, waist circumference, body mass index)	(+) in cardiorespiratory fitness (−) in physical activity level and body composition	Partial (cardiorespiratory fitness, strength, stability)
Feito et al. (2019)	8 weeks	18 adults (25-27 years)	2 EG § CG	3 times/week of aerobic (running, rope jumping), body weight strength exercises (pull-ups, squat), and weightlifting exercises (jerk, kettlebell swing)	Total training session duration Body composition (arm lean mass, arm fat mass, trunk lean mass, trunk fat mass, leg lean mass, leg fat mass, waist circumference, body mass index Glucose control (Fasting Plasma Glucose)	(+) in total training session duration (−) in body composition and in glucose control	Partial (strength, cardiorespiratory fitness, agility)
Heinrich et al. (2014)	8 weeks	23 adults (26.8 ± 5.9 years)	2 EG § CG	2 times/week of aerobic exercises (rowing ergometer) combined with nine resistance training exercises (air squat, front squat, overhead squat, press, push press, push jerk, deadlift, sumo deadlift, high pull, medicine ball clean)	Body composition (body fat percentage, body mass index) Cognitive-psychological performance (exercise enjoyment scale)	(−) in body composition (+) exercise enjoyment	Partial (strength, cardiorespiratory fitness, agility)

Note: (+) = significant positive changes of INT group compared to other training interventions; (=) = similar significant changes of INT group respect to other training interventions; (−) = no significant changes between interventions, § = Intervention based on INT approach.

*EG* Experimental Group, *CG* Control Group

The main finding of this narrative review was that INT intervention is effective to improve both health-related and skill-related fitness components in individuals with obesity, independently from training duration and participants’ age. On one hand, balance, postural control, and movement competence appear to be better improved by INT than a traditional training regimen. On the other hand, body composition and cardiorespiratory fitness exhibited similar enhancements compared to a common intervention. In addition, it seems that motor adaptations obtained by INT were slightly preserved during a subsequent detraining period.

## Discussion

### Study population: younger versus older individuals

INT appears to be effective in ameliorating postural control in children and adolescents with obesity ranging from seven to sixteen years old (*N* = 5 studies). In this regards, seven-year-old children demonstrated significant changes in Center of Pressure variables (i.e., sway area, mean velocity, antero-posterior velocity, medio-lateral velocity) and dynamic balance score measured with the Star Excursion Balance Test following a training program focused on INT approach (e.g., mini hurdle jumping, agility cone drills, single-leg balance, marching in place, single-leg squat) [30]. Similarly, Molina-Garcia and colleagues demonstrated that 10-year-old participants who followed a movement-quality exercise intervention composed of exercise to improve awareness of movement pattern, mobility, stability and strength combined with multi-games exercises, significantly improved their posture in various body segments (e.g., lower limb angle and plumb-tragus distance), functional performance in the deep squat and active leg raise patterns, as well as maximum forefoot force support, while maintaining their foot and pelvic alignment during the walking pattern when compared to a conventional lifestyle intervention [31–33]. Conversely, in adolescents (age range from 13 to 16 years), an INT intervention composed of a goal-directed activity (e.g., throwing at targets, emptying boxes, walking, running) and team-based games compared to a video games program using Nintendo Wii gaming system induced similar ameliorations in muscular strength and in both aerobic and anaerobic fitness profile without between-groups differences [34].

Although this type of training has been developed especially for a younger population with the aim to minimize the injury risk during sport activities, thanks to a better movement symmetry and “motor awkwardness” [23, 28, 35], there is a plethora of studies that demonstrated the effectiveness of INT also in adulthood by enhancing both health and skill-related physical fitness components. Moreover, INT improved also cognitive and psychological performance (e.g., selective attention, cognitive flexibility, motivation, self-efficacy, vitality, enjoyment) with contrasting results on body weight change or fat mass reduction [36–49].

### Duration of intervention: short and long-time training effects outcomes

INT training mode induced significant adaptation responses overtime ranging from a short, to a mid-long-time period people with obesity. In two studies, a 3-week training duration was sufficient to improve cardiorespiratory fitness, respiratory muscles strength, quality of life and postural stability [41, 46]. In this regard, Bezzoli et al., tested the efficacy of a 3-weeks multidisciplinary rehabilitation program composed by one daily 30-min conditioning session on a bike ergometer (intensity of 60–70% heart rate maximum) combined with motor control exercises and rib cage mobility. The Authors demonstrated significant improvements in pulmonary function (Forced Vital Capacity, Maximal Voluntary Ventilation, Maximal Expiratory Pressure), quality of life (Patient Specific Function Scale) and cardiorespiratory fitness (6 minute walking test) [41]. On the same line of evidence, also Maffiuletti and colleagues showed that a short-time intervention period (3 weeks) targeting body weight reduction strategies as energy restricted diet, psychological counseling and physical activity combined with balance and proprioceptive exercises improved trunk postural sway and time of balance maintenance in individuals with obesity [46]. Similarly, Guzmán-Muñoz et al. showed that a 4-week training program based on neuromuscular stimuli (e.g., mini hurdle jumping, agility drills, single-leg balance, marching in place, tandem walk, and single-leg squat) enhanced static (e.g., mean velocity and sway area center of pressure) and dynamic postural variables (e.g., maximal anterior, postero-lateral and postero-medial reach distances during a monopodalic stance) [30]. A period with the same duration appeared to be effective in ameliorating body balance detected through the single leg test and the Star Excursion Balance Test following a specific training protocol (4 week/sessions for 45 min) conducted with adult individuals who were treated with a sleeve gastrectomy [47].

For what concern mid-time effects ranging from six to fourteen weeks of intervention duration, there is evidence promoting positive effects on physical fitness outcomes (i.e., muscular strength, body balance, flexibility, cardiorespiratory fitness) and negligeable changes in anthropometry and body composition both in adults [42, 44] and adolescents [31–34].

Lastly, when dealing with long-time interventions, Batrakoulis et al. demonstrated that an INT program conducted over 10 months was able to increase daily energy expenditure, reduce fat mass, improvement muscular strength and cardiovascular performance in forty-nine individuals with obesity [37]. The same research group highlighted that a progressive INT based protocol (e.g., squat, hinge, lunge, push, pull, carry, rotation, plank exercises) using portable tools (e.g., suspension, belts, balance balls, kettlebells, medicine balls, battle ropes, stability balls, speed ladders, foam rollers, elastic bands) preserved muscular strength, aerobic fitness, flexibility, bone mineral density and psychological regulation (e.g., wellbeing, vitality) in inactive females with obesity [39, 40]. Moreover, positive findings were also detected following eighteen weeks of weight loss combined with an INT program in obese elderly adults, promoting significant improvements in physical function tested via Short Physical Performance Battery, abdominal visceral (VAT) and thigh intermuscular adipose tissue (IMAT), when compared to individuals who followed a traditional physical activity program combined with educational lifestyle workshops [49]. Finally, La Scala Teixeira et al. demonstrated that an INT protocol with a duration of 30 weeks improved cardiorespiratory fitness, whereas controversial changes in fat mass, body weight and lean mass were found. However, waist circumference showed a significant reduction overtime when compared to other training interventions (e.g., interdisciplinary therapy and interdisciplinary education) [45].

### Cardiorespiratory fitness outcome

Cardiorespiratory fitness was investigated in six studies [33, 34, 37, 41, 45, 48] reporting significant improvements following a specific neuromuscular training protocol. Batrakoulis and colleagues (2018) showed positive changes in VO2_max_ from baseline (26.1 ± 4.4 mL/kg/min) to post-training (33.1 ± 4.8 mL/kg/min; *p* < 0.001) following a INT protocol (e.g., high-intensity whole-body multi-joint movements in a circuit training modality respecting a heart rate maximum major of 65%) with a concomitant significant reduction in blood lactate concentration during the entire training phase (i.e., from week one to week forty) [37]. On the same line of evidence also Bezzoli et al., reported significant improvements in distance covered during the six-minute walking test (403.4 ± 158.4 m pre-training versus 464.6 ± 131.6 m post-training) in obese individuals who performed neuromotor exercises combined with conditioning sessions performed at 60–70% of their maximum heart rate [41]. Notably, also La Scala Teixeira and colleagues observed a significant main effect of time on VO2_max_ (*F* = 12.441, *p* = 0.001) in individuals who performed different functional training circuits composed by free weights, elastic bands and bodyweight movements (e.g., upright row with sumo squat, dumbbell fly with pelvic elevation, elastic trunk rotation, front raise with side lunge, suspended row, knee flexion with elbow flexion, trunk lateral flexion, single leg balance with eyes closed) (*p* = 0.014) [45]. Conversely, three studies did not observe between-group changes in this specific outcome following a INT program [33, 34, 48].

### Muscular strength outcome

Concerning the effects of INT on muscular strength, five out of seven studies reported significant improvements. Specifically, the upper and lower-body strength assessed with different procedures (e.g., one repetition maximum in vertical chest press, one repetition maximum in supinated lat pull-down, one repetition maximum in arm press, isometric handgrip strength test, one repetition maximum in horizontal leg press, one repetition maximum in seated leg extension, one repetition maximum in leg curl, sit to stand test, standing long jump test, isometric knee extensor test, isometric ankle plantar-flexors and dorsi-flexor test) showed positive changes in individuals with obesity in favor to an INT approach [33, 37, 38, 40, 42]. Two studies [34, 48] found similar improvements overtime without differences between training interventions (INT protocol versus control) for both upper and lower-body segments. On one hand, it could be speculated that these controversial results might be due to a reduced adherence to the training program, or to the equipment used (e.g., elastic bands, dumbbells, suspension belts, kettlebells, medicine balls, battle ropes), but on the other hand the duration of each training program (e.g., from eight weeks to ten months) induced a better movement pattern learning and muscle recruitment leading to positive changes in muscular strength [50, 51].

### Flexibility outcome

In three studies, joint range of motion showed significant changes between groups [38, 40, 48]. In detail, Batrakoulis et al. demonstrated remarkable variations (*p* < 0.001) in sit and reach test and in glenohumeral internal rotation in favor to INT group respect to control group. For what concern ankle dorsi-flexion and shoulder extension test there is trend in reaching a significant difference (*p* = 0.057) [40]. Moreover, the same research group demonstrated that an INT program performed with a different frequency (e.g., one day/week, two days/week, or three days/week) was able to induce significant improvements compared to what observed in the control group (*p* = 0.025) [38]. Specifically, one day/week was more effective to ameliorate ankle dorsiflexion and shoulder flexion; two days/week induced significant ameliorations in passive range of motion in ankle dorsiflexion, hip flexion and shoulder flexion. Finally, three days/week promoted a better flexibility (*p* < 0.001) in ankle dorsiflexion, knee flexion, hip flexion and shoulder flexion [38]. Lastly, the study by Cambiriba and colleagues found that the flexibility detected through the Wells bench test reached similar changes in the experimental group who followed a training protocol focused on INT principles when compared to control group [48].

### Body balance, postural control, and functional performance outcomes

Several studies have reported positive changes in both static [42, 46, 47] and dynamic balance [30, 47], measured in different stances, after a period of INT, underlying the importance of a neuromuscolar training for stimulating proprio and exteroception in individuals with obesity. On the same line of evidence, the center of pressure sway area and velocity [30], combined the maximum force sustained by the medio-lateral forefoot, the lower limb angle, pelvic tilt and the plumb-tragus alignment significantly improved (*p* < 0.05) thanks to neuromotor exercises whose aim was to preserve an optimal spine position, self-awareness of movement patterns, core muscles stabilization and muscular strength [31, 33]. Functional performance has also been shown to improve with INT intervention, with five studies reporting a significant increase in the Functional Movement Screen total score [38, 39, 42] and single scores in the Hurdle Step (*p* = 0.0036), Active Straight Leg Raise (*p* = 0.0003) and Deep Squat (*p* = 0.004) movement patterns [32, 42]. Moreover, also Santanasto and colleagues demonstrated a significant amelioration in functional capacity after an INT protocol using the Short Physical Performance Battery, with scores increasing from 9.7 at baseline to 10.3 arbitrary units at the end of the intervention period in older people with obesity [49]. Taken together, all these findings provides evidence that individuals with obesity may benefit from an INT protocol focused on motor control, articular stability and movement competence in ameliorating their body balance and postural control.

### Cognitive performance outcome

Regarding the effects of INT on cognition, three studies showed beneficial adaptations on cognitive and psychological performances [36, 39]. In detail, psychological distress scores detected using a specific questionnaire (i.e., General Health Questionnaire -12) significantly decreased by 68%, vitality score increased by 53% (*p* = 0.001) and finally also the introjected regulation, intrinsic regulation and identified regulation significantly improved throughout the five-month period (*p* = 0.001; *p* = 0.004, *p* = 0.001). During the subsequent 5 month-detraining period, these results were attenuated, but not completely lost when compared to baseline scores [39]. As for neurological functions, the brain-derived neurotrophic factor had a significant enhancement in both active and inactive obese women, as well as the executive function performances following a specific INT training regimen [36]. Last but not least, a specific neuromuscular training program presented significant changes overtime in exercise enjoyment (*p* = 0.002) compared to a common exercise protocol [44].

### Body composition and anthropometry outcomes

With respect to body composition indicators INT appeared to induce similar changes compared to traditional training. Three evidences reported a remarkable reduction in visceral fat (−15.1%, *p* = 0.004), percent body fat (−7.2, *p* = 0.001), intermuscular adipose tissue (−23.6, *p* = 0.001), subcutaneous tight fat (−12.3%, *p* = 0.002), body weight (−5.5%, *p* = 0.002) and BMI (−5.2%, *p* = 0.002) after 12 month of INT [49]. On the same line of evidence, Batrakoulis and colleagues highlighted significant improvements by 1.9% in bone mineral density and 1.5% in bone mineral content [40] with positive changes also in BMI, lean mass and waist circumference [37]. Notably, these ameliorations were mitigated following a 5-month detraining observing a slight weight regain and body composition profile (body mass, *p* = 0.003; BMI, *p* = 0.004; waist circumference, *p* = 0.001; hip circumference, *p* = 0.015; resting metabolic rate, *p* = 0.001) without reaching baseline levels [37]. By contrast, non-significant interactions and between-group differences were observed in BMI [41–45], fat mass percentage [41–45], lean mass [43, 45], waist circumference [41–43, 49], body weight [44], muscle density [49] and hip circumference [41] suggesting similar changes regardless of the exercise nature (e.g., INT or traditional exercise resistance training).

In summary, these studies analyzed the contribution of the INT regimen on physical fitness and body composition to counteract obesity. In fact, it is well documented that a multidimensional exercise program targeted on the mixture of various fitness components are able to achieve whole-body postural improvements, muscular strength and cardiorespiratory fitness thanks to a more efficient neural plasticity underlying a more proficient motor skill learning [52] and movement competency [26]. An exercise intervention that incorporates INT principles (i.e., health and skill-related variables) focused on dynamic mobility, stability, fundamental movement pattern correction, cardiovascular and cognitive stimuli, may help to develop neural adaptations, new motor units recruitment, intermuscular coordination and strength gains [53, 54]. These findings suggest that the improvements due to INT contribute to better quality of movement and postural control during daily-life activities. This is significant, as increased body weight in known to lead to balance deficits compared to normal-weight individuals [55]. Moreover, an INT regimen characterized by high-intensity body-weight circuit training exercises displayed significant ameliorations in skeletal muscle oxidative capacity, a delay muscle fatigue perception and a cardiovascular endurance improvements [56].

Despite INT exhibits similar results compared to a common intervention in some outcomes (e.g., body composition, cardiorespiratory fitness), INT seems to provide other beneficial implications for obesity as a major daily energy expenditure, and lean mass percentage, bone mineral density, and psychological wellbeing (e.g., enjoyment, vitality, distress regulation and executive function). Nevertheless, body weight, fat mass percentage, waist circumference and BMI tended to have a similar behavior as the other traditional training protocols. From a speculative view point, these body composition findings suggest the key role that a supervised nutritional management may have, in combination with a regular physical activity (preferably based on neuromuscular stimuli) to obtain a more pronounced fat loss and weight reduction [57–59].

It is interesting to note that training interruption is a recognized problem that occurs during weight loss interventions, with a frequent tendency to regain body mass reaching values similar to pre-intervention [60]. Noteworthy, an INT protocol appeared to be adequate to mitigate the negative effects of training cessation (i.e., five months) showing slower decrements in both health-related (i.e., cardiovascular, strength, flexibility) and skill-related (e.g., balance, motor coordination) outcomes [37, 38, 40]. Moreover, as INT was shown to improve enjoyment with respect to a traditional training program [44], an INT protocol may be suitable to prevent training interruption.

Lastly, it is worth noting that regular physical exercise that incorporates INT principles is a safe and injury-free option for both children and adults. This is likely due to the multicomponent, high-intensity and intermittent nature of this training, which includes neuromuscular and cognitive tasks enhancing muscular performance and motor coordination, thereby reducing the risk of injury [38].

From a practical perspective, this narrative review lays the initial foundation to promote the usefulness of INT to treat obesity in a population with different age (e.g., younger and older) and anthropometry. These evidence on INT allows fitness coaches, therapists and practitioners to consider INT principles a feasible alternative to the most common exercise intervention used within obesity. Taking this into account, a customized exercise training and a structured nutritional approach may display an important role in supporting individuals with various health conditions, including physical disabilities [61], obesity [62], cancer [63] and diabetes mellitus [64].

Nevertheless, some limitations of the present study should be recognized. First, the narrative nature of the review (as compared to systematic approach with meta-analysis) prevents us to depict firm conclusions about the efficacy of prescribing INT protocols for individuals with obesity. Second, the limited number of electronic databases considered during the searching process may have contributed to limit the discussion of the results. Third, studies utilizing telemedicine approaches were not considered in the present study, although this modality seems to be a new frontier gaining even more attention into research and clinical setting in obesity management [65].

In conclusion, this review highlights the beneficial role of INT programs in improving physical fitness in both younger and older individuals with obesity, with significant ameliorations over short, medium and long time period. The most INT outcomes used in the analyzed studies were cardiorespiratory fitness, muscular strength, balance, postural control and body composition, with significant remarkable improvements following a specific training intervention based on INT approach targeting both health-related and skill-related physical fitness components. However, BMI, fat mass percentage and waist circumference showed comparable improvements with respects to traditional training exercises. Taken together, these findings support the effectiveness of INT protocols in ameliorating health-related and skill- related physical fitness components. Therefore, fitness coaches and therapists may consider INT as feasible option when working with outpatients with obesity.
